# Nanodiamond Influence on the Nucleation and Growth of YBCO Superconducting Film Deposited by Metal–Organic Decomposition

**DOI:** 10.1021/acs.cgd.3c00607

**Published:** 2023-07-15

**Authors:** Valentina Pinto, Angelo Vannozzi, Giuseppe Celentano, Massimo Tomellini, Alexander Meledin, Silvia Orlanducci

**Affiliations:** † Superconductivity Laboratory, FSN-COND, ENEA, Via E. Fermi 45, 00044 Frascati (Rome), Italy; ‡ Department of Chemical Sciences and Technologies, Via della Ricerca Scientifica, Tor Vergata University, Rome 000173, Italy; § Central Facility for Electron Microscopy, RWTH Aachen University, Ahornstraße 55, 52074 Aachen, Germany

## Abstract

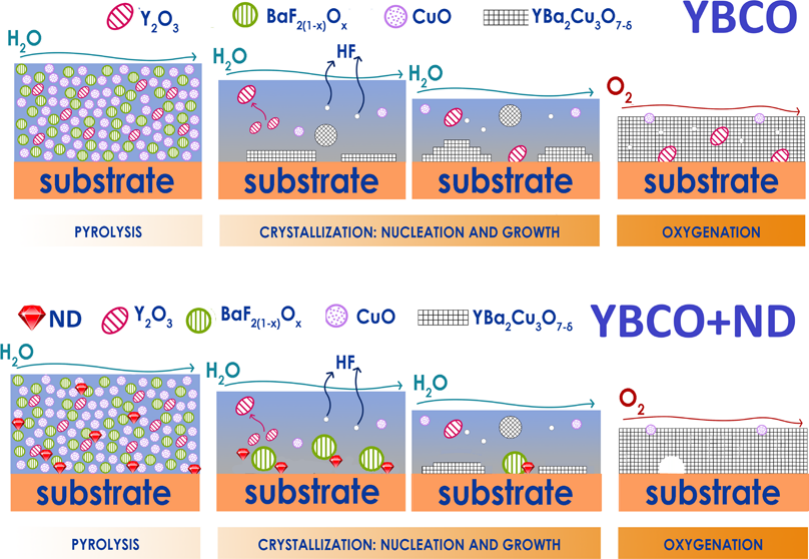

It was recently shown that the introduction of nanodiamond (ND) into a superconducting metal–organic deposited YBa_2_Cu_3_O_7−δ_ (YBCO) film produces an increase in critical current density in self-field conditions (*B* = 0 T). Such improvement appears to be due to the formation of denser and smoother films than the samples deposited without ND. This paper presents the work done to understand the role of ND during YBCO nucleation and growth. A detailed study on YBCO+ND films quenched at different temperatures of the crystallization process was carried out. Results showed that the reaction responsible for YBCO production appeared effectively affected by ND. In particular, ND stabilizes one of the YBCO precursors, BaF_2(1–*x*)_O_*x*_, whose conversion into YBCO requires a prolonged time. Therefore, the YBCO nucleation is slowed down by ND and begins when the experimental conditions favor both thermodynamically and kinetically the formation of YBCO along the *c*-axis. This effect has important implications because the growth of a highly epitaxial *c*-axis YBCO film enables excellent superconducting performance.

## Abstract 2

The nucleation and growth of a YBCO epitaxial film can be delayed by the presence of nanodiamond (ND). Since YBCO nucleation occurs at a higher temperature, the *c*-axis grain formation is favored from both a thermodynamic and kinetic point of view. This effect can explain the improvement of superconducting properties observed in a YBCO+ND film.

## Introduction

The use of superconducting materials for power application was envisaged since their discovery in 1911.^1,2^ Penetration of the magnetic field above the lower critical field *H*_c1_ as flux quanta (fluxons) inside the superconductor and their resulting motion due to the Lorentz force, in the absence of defects able to pin them, produce dissipation and no critical current can be observed. Therefore, pinning fluxons is a crucial issue for the practical use of all superconducting materials. Intrinsic pinning due to lattice defects such as grain boundaries, impurities, dislocations, point defects, and so on are spontaneously generated during material processing.^3,4^ However, more effective pinning can be obtained through the introduction of the so-called artificial pinning centers (APCs). In this way, the controlled size and shape of APCs can be engineered, depending on the operation conditions in which the superconductor is designed to be used.^3,5^

Rare-earth cuprate materials *RE*Ba_2_Cu_3_O_7-δ_ (*RE*BCO, in which *RE* is Y or rare earth) are widely studied as epitaxial films for the production of superconducting tapes (coated conductors) for power applications. Two main routes are used for film deposition, namely, pulsed laser deposition (PLD) and metal–organic decomposition (MOD). While the former technique is well established and widely used for industrial production of *RE*BCO tapes, the latter is considered very appealing due to the inexpensive setup, basically consisting of the deposition of the precursor solution and a conversion heat treatment. However, while a high amount of defects are typically generated by means of the PLD technique, chemical solution deposition (CSD) of *RE*BCO films by MOD leads to comparatively low-defect films showing poor intrinsic pinning. Therefore, APC introduction in MOD-derived *RE*BCO films is necessary to increase their performances in order to be able to compete with PLD-grown films. The introduction of APCs in *RE*BCO films by PLD is easily carried out by adding the desired second phase to the *RE*BCO target (e.g., BaZrO_3_, BaHfO_3_, Ba_2_YNbO_6_, Ba_2_YTaO_6_).

The introduction of APCs in the *RE*BCO matrix by MOD can be obtained according to two main approaches, i.e., adding either the precursor of the second phase (in situ) or preformed nanoparticles (NPs) (ex situ) to the *RE*BCO precursor solution. The former approach is carried out by adding the desired precursor or an excess of one element, such as Y or Gd, to the precursor solution. Metal ions exceeding the 123 *RE*BCO stoichiometry will form other phases.^6−8^ The latter approach includes the preparation of preformed NPs, which are dispersed in the precursor solution. The main advantage of using preformed NPs is the possibility of easily customizing the size and concentration of the precursor solution. Among the proposed NPs used as APCs, BaMO_3_ (M = Zr, Hf, Sn) can be mentioned.^9−11^ Nanodiamond (ND) was proposed as an APC in metal–organic deposited YBCO films for the first time a few years ago.^12,13^ Detonation NDs are easily produced with a controlled size below 10 nm, suitable for zero-dimensional defect generation. Our first experiments showed that ND addition to YBCO solution positively affects both film morphology and superconducting properties. YBCO+ND films are generally more compact, smoother, and denser with higher critical current density, *J*_c_. By *I*–*V* analyses, self-field *J*_c_ values (*B* = 0 T) of 4 and 18 MA·cm^–2^ are obtained for YBCO+ND at 77 and 10 K, respectively, compared with *J*_c_ values of 2.3 ± 0.9 and 13 ± 1.2 MA·cm^–2^ of pure YBCO. The higher *J*_c_ in self-field condition measured in YBCO+ND samples is likely due to a better grain coalescence and higher film density, which leads to an increase of the percolation paths in the film.

Moreover, the *J*_c_ (*B*, *T*) curves for the set of samples demonstrated that ND has not a clear influence on vortex pinning, likely due to the ND concentration used in YBCO solution, namely, ≤ 5 mol %, which is very low in comparison with NP concentration studied by other laboratories. In fact, the introduction of NPs such as BaMO_3_, ZrO_2_, and Ba_2_YTaO_6_, with concentrations in the range of 5–32 mol % produced a significant pinning increase. Besides, the effect on *J*_c_ in self-field condition is dependent on both the nature and concentration of NPs,^9−11,14−19^ although increased self-field *J*_c_ is observed only in a few studies.^10,19^

Conversely, some articles reported that YBCO films prepared by MOD and using Au, Ag, and Pb as dopants showed an improvement in morphology along with higher self-field *J*_c_ values. In those cases, when a metallic dopant is used, the formation of intermetallic phases is likely responsible for chemical potential changes of *c*- and *a*-axis nucleations favoring the *c*-axis orientation.^20−23^ In analogy to such studies, it may be assumed that the addition of ND is beneficial to *c*-axis film nucleation during the crystallization step. However, in the case of carbon-based nanomaterials (CBNs), the intermetallic phase formation can be excluded, so a different mechanism (or influence) should be hypothesized.

Therefore, in the present paper, we report the results of experiments designed and performed with the aim of clarifying the role of ND in promoting YBCO nucleation and growth. The microstructural properties of YBCO films produced with or without ND and quenched at different steps of the thermal treatment were characterized, shedding light on the effect of ND on the reaction of YBCO formation. Based on the obtained results, an explanation of the improvement in film performances is provided.

## Experimental Section

### Materials

Ammonium hydroxide ultrapure solution (NH_4_OH, MW 35.05 g·mol^–1^, J.T. Baker, Ultrex, 20–22%), barium (II) trifluoroacetate hydrate (Ba(CF_3_CO_2_)_2_·*x*H_2_O, MW 363.37 g·mol^–1^ (anhydrous basis), Alfa Aesar), copper (II) acetate (Cu(CO_2_CH_3_)_2_, MW 181.63 g·mol^–1^, Sigma-Aldrich, trace metals basis, 99.99%), methanol (CH_3_OH, MW 32.04 g·mol^–1^, Sigma-Aldrich, for HPLC gradient grade, ≥99.9%), nanodiamond (ND) powder (produced by detonation from International Technology Center, ITC, with purity > 98% and 4–5 nm primary particle size), propionic acid (CH_3_CH_2_COOH, MW 74.08 g·mol^–1^, Sigma-Aldrich, ACS reagent, ≥99.5%), yttrium (III) acetate hydrate (Y(CH_3_CO_2_)_3_·*x*H_2_O, MW 266.04 g·mol^–1^ (anhydrous basis), Sigma-Aldrich, 99.9%), strontium titanate (SrTiO_3_ (STO), (001) single crystals, size: 7.5 × 7.5 mm^2^, Wollemi Technical Incorporation and MaTecK (Material Technologie & Kristalle GmbH)), silicon ⟨100⟩ wafer (NanoVision s.r.l. (Gambetti Kenologia)) were used.

### Equipment

All labware used in the experiments was soaked in diluted HNO_3_ overnight and then rinsed with ultrapure water. Ultrapure water (18.2 MΩ·cm at 25 °C) was produced by the Synergy UV Remote system integrated with the Elix module, supplied by Millipore. Solution homogenization, substrate cleaning, and nanodiamond dispersion were carried out using Elmasonic S30H, Elma GmbH, and Sonica 2200, Soltec, ultrasonic baths. An ultrasonic homogenizer probe, Bandelin SONOPULS HD2200, was employed for colloidal dispersion preparation adopting the following conditions: 40% power, 20 min each run. A Rotavapor R-215 B.U.CHI was used as a rotary evaporator during YBCO precursor solution preparation. The precursor solution was deposited by spin coating using the spinner WS-400BZ-6NPP/LITE supplied by Laurell Corporation. For the thermal treatment, a homemade tube furnace with a furnace body length of 150 mm and a Eurotherm 2048 Temperature controller/programmer for the control of thermal cycles was used. A quartz tube with an inner diameter of 2.5 cm was employed. The furnace was coupled with gas flowmeters (300 VUE, Teledyne, Hastings Instruments) to regulate the atmosphere. Humid gases were obtained by bubbling the carrier gas through three Drechsel flasks partially filled with ultrapure water kept at 21 °C (corresponding to a saturated vapor partial pressure of 25 mbar).

### Methods

#### YBCO Film Synthesis

##### Preparation of YBCO Precursor Solution

In a typical experiment, a pure YBCO low-fluorine solution, 0.2 M referred to as [Y^3+^], was prepared with yttrium (III) acetate hydrate (1 mmol), barium (II) trifluoroacetate hydrate (2 mmol), and copper (II) acetate (3.1 mmol). A small excess of copper acetate was used in consideration of the copper loss possibly occurring during the pyrolysis step.^24^ Precursors’ salts were separately dissolved in excess methanol (MeOH) in three different beakers employing an ultrasonic bath for 5 min. Then, exact volumes of propionic acid, with a total of 5 mL, were added to each solution that was further sonicated for 5 min. Finally, 0.5 mL of NH_4_OH was added to the Cu solution to favor metal solubilization. At the end of this procedure, the salts appeared completely dissolved and the three solutions were merged in a single beaker and underwent another 10 min of sonication. Thereafter, the solution was quantitatively transferred in a round-bottom flask using MeOH for beaker cleaning. Then, a rotary evaporation process was performed at 58 °C/306 mbar and 75 °C/153 mbar to eliminate both MeOH and water content. The solution was transferred and stored in an amber vial in a flowing nitrogen desiccator.

##### Nanodiamond Colloidal Dispersion and YBCO+ND Precursor Solution

Detonation ND with 4–5 nm primary particle size diameter and 30 nm average agglomerate size was used for the preparation of the colloidal dispersion. ND, 6 mg accurately weighed, was dispersed in 20 mL of propionic acid using first an ultrasonic bath for 4 h. After overnight sedimentation, the supernatant was separated from the solid residue and treated with a more powerful sonicator (the ultrasonic homogenizer probe) at room temperature for 6 h.

At the end of the sonication treatment, the ≤10 nm size of nanoparticles in the dispersion was verified by scanning electron microscopy (SEM) and atomic force microscopy investigations performed on samples prepared by depositing 10 μL of ND dispersion on the Si wafer through spin coating (3000 rpm, 60 s) and drying for 15 min at 130 °C in air.

A rough estimation of ND concentration ≤ 0.01 M in the dispersion was calculated on the basis of the recovered precipitate.

The YBCO+ND precursor solution was prepared by modifying the usual procedure and replacing pure propionic acid with ND dispersion. The amount of C introduced in YBCO solution in the form of ND can be approximately considered to be ≤ 5 mol % with respect to Y^3+^ concentration.

##### YBCO Film Deposition

The precursor solution was spin-coated for 60 s at a spinning rate of 3000 rpm on the STO substrate. In standard conditions, films were pyrolyzed in a quartz tube furnace with flowing oxygen (0.94 L·min^–1^, humid for *T* > 100 °C) up to 480 °C using the following heating ramp rate: 10 °C·min^–1^ (up to 90 °C), 2.5 °C·min^–1^ (in the range of 90–200 °C), 1.2 °C·min^–1^ (in the range of 200–300 °C), 10 °C·min^–1^ (in the range of 300–480 °C), and 10 °C·min^–1^ (during the cooling step).

A second treatment, named “firing”, was then performed to promote YBCO crystallization and oxygenation. First, the sample was heated with a rate of 10 °C·min^–1^ to the dwell temperature of 830 °C and kept for 50 min in a humid mixture of nitrogen and oxygen (flow rate N_2_: 2.83 L·min^–1^, O_2_: 1.15 × 10^–3^ L·min^–1^) and further 10 min in the same dry mixture (crystallization step). Subsequently, the film was cooled down to 450 °C with a rate of 10 °C·min^–1^, kept at this temperature for 15 min in dry oxygen (0.94 L·min^–1^) for the oxygenation step and then cooled to room temperature with the same ramp rate. A final film thickness of 78 ± 3 nm was obtained.

##### Films Deposition for the Nucleation and Growth Study

**Figure 1:**
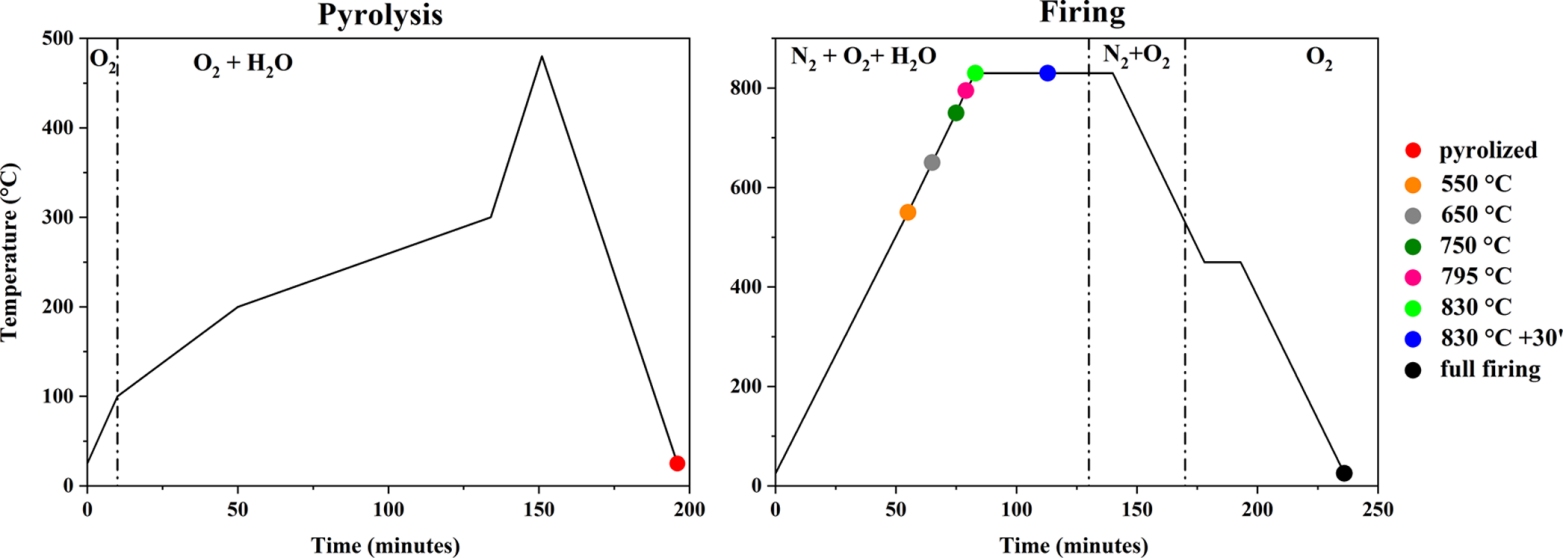
Schematic representation of the pyrolysis and firing steps of the thermal treatment. The samples produced for studying the evolution of film composition are evidenced as colored dots.

YBCO+ND thin films were deposited on the STO substrate by spin coating, using the standard pyrolysis and firing conditions optimized for pure YBCO films. In order to clarify the influence of ND on YBCO nucleation, a specific experiment aimed at studying the structural and chemical evolution of propionate-based low-fluorine YBCO precursors during the conversion thermal treatment was carried out. In particular, a set of partially converted samples was prepared: one sample pyrolyzed at 480 °C, six samples quenched to different temperatures during the firing treatment (i.e., quenched at 550, 650, 750, 795, 830 °C and one sample treated at 830 °C for 30 min before quenching) and another one subjected to the complete thermal process necessary to promote full YBCO conversion. The same experiment was performed on both YBCO+ND and pure YBCO films. The latter ones were used as a reference. A final thickness of about 80 nm was obtained in all cases. A schematic representation of the thermal treatment and of the produced samples is reported in Figure 1. In our previous article,^13^ problems with YBCO+ND solution stability were observed despite the ND concentration in YBCO solution being estimated to be very low, [ND] < 5 mol %. However, it was verified that samples prepared with fresh solution and within the same day show comparable properties. Therefore, the YBCO+ND samples necessary for the nucleation study were pyrolyzed on the same day with a precursor solution prepared no more than 12 h before.

#### Film Characterization

##### Morphology

Optical microscopy was employed to investigate film surface morphology after the thermal treatment. A Nikon SMZ18 stereomicroscope and a Leitz–Wetzlar Metalloplan metallographic microscope were used for this purpose.

SEM images were acquired by a Gemini LEO 1525 field emission high-resolution SEM with 10–20 kV accelerating voltages and an in-lens high-resolution annular detector.

##### Structural Analysis

The X-ray diffraction (XRD) technique was employed for the structural properties and texture analyses of pure and composite YBCO films. A Rigaku Geigerflex diffractometer with Cu Kα radiation in Bragg–Brentano configuration was used for the acquisition of X-ray θ–2θ and ω-scans.

YBCO+ND films were also analyzed by transmission electron microscopy (TEM). For TEM investigations, a cross-sectional lamella was produced by a focused ion beam technique, employing an FEI Dual Beam Helios NanoLab system. The cross-sectional lamella was taken from the strips used for transport analyses in order to correlate the information gained from TEM with superconducting properties. The annular dark-field and high-angle annular dark-field scanning transmission electron microscopy (ADF and HAADF-STEM) imaging and energy-dispersive X-ray spectroscopy (EDX) were performed using an FEI Titan electron microscope, equipped with a Cs-aberration corrector for the probe-forming lens and a “Super-X” wide solid angle EDX detector operating at 200–300 kV acceleration voltages.^25,26^

##### Superconducting Properties

The zero-resistance critical temperature, *T*_c_, was assessed through *R*(*T*) measurements performed by d.c. electric measurements in the four-probe configuration with a 2420 Keithley current source meter (used current 100 μA) and a 2182 A Keithley nanovoltmeter.

## Results

The fully processed YBCO and YBCO+ND samples showed morphological, structural, and superconducting properties in line with our previously reported samples.^13^ In particular, the recorded XRD patterns showed only YBCO (00) diffraction peaks and revealed the good *c*-axis-oriented grain structure in both pure YBCO and YBCO+ND films. As already reported,^13^ no significant differences were detected in the fully converted films as regards several parameters such as the full width at half-maximum (FWHM) calculated from (005) YBCO ω-scan, *c*-axis lattice parameter, and intensity ratio, i.e., similar crystallinity.

**Figure 2:**
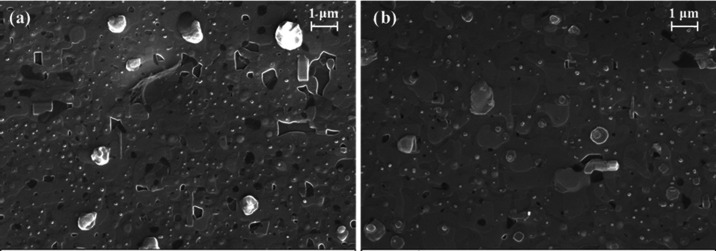
SEM images at the same magnification of YBCO (a) and YBCO+ND (b) films deposited simultaneously during the same thermal treatment. The scale bar corresponds to 1 μm.

SEM analyses confirmed an improvement in film morphology when ND was added. In fact, pure YBCO showed several uncovered substrate regions, while the YBCO+ND film displayed only a few pores. Further, in the latter, a lower amount of sub-micrometric particulates occurred (Figure 2).

**Figure 3:**
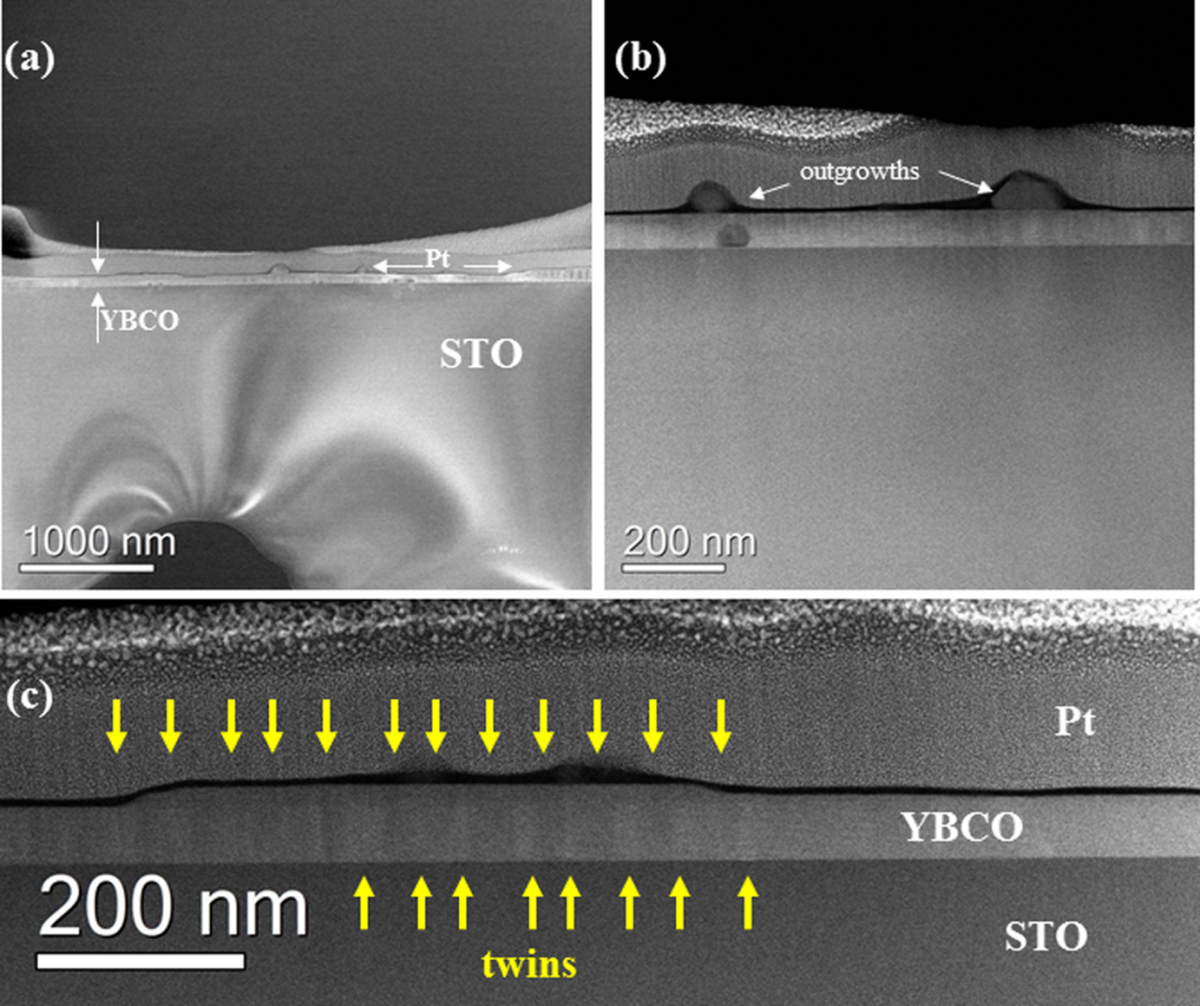
(a) HAADF-STEM overview of the YBCO+ND film deposited by the standard process (taken along the [100]/[010]YBCO zone axis), (b) some outgrowths segregated at the surface, and (c) high density of twin boundaries (highlighted by yellow arrows).

The effect on the YBCO structure due to ND addition was further investigated through the TEM analysis of the YBCO+ND film deposited using the standard thermal treatment. In Figure 3, the HAADF-STEM cross-sectional overview of the film on the STO substrate is shown. An ordered and dense YBCO structure was evident, along with a high density of twin boundaries (highlighted by yellow arrows in Figure 3c). The surface of the layer was decorated by several secondary phase particles.

**Figure 4:**
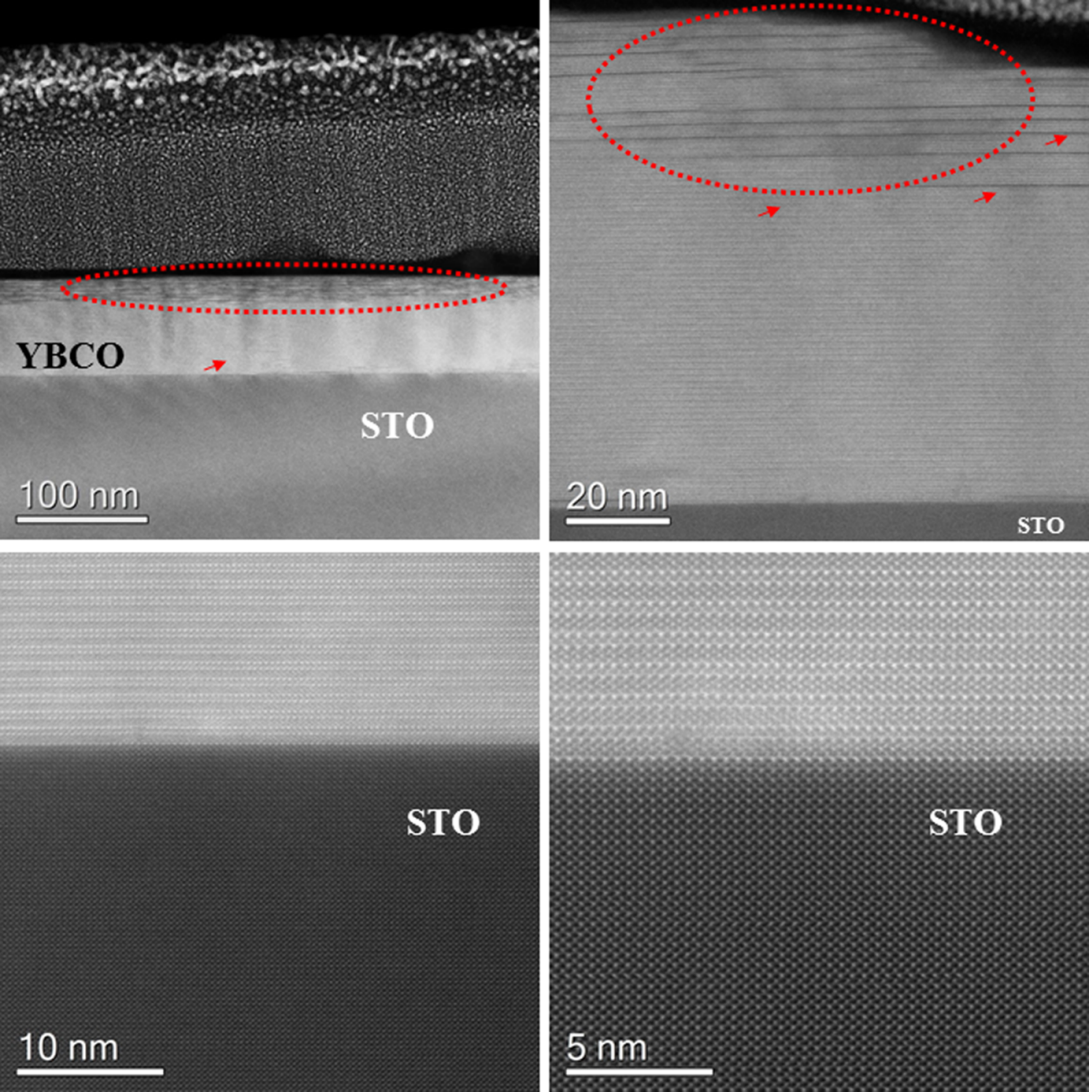
HAADF-STEM view of areas with a high density of stacking faults, marked by red arrows and a dotted line, present at the surface (upper panel). The sharp interface between YBCO and STO is shown in the lower panel.

**Figure 5:**
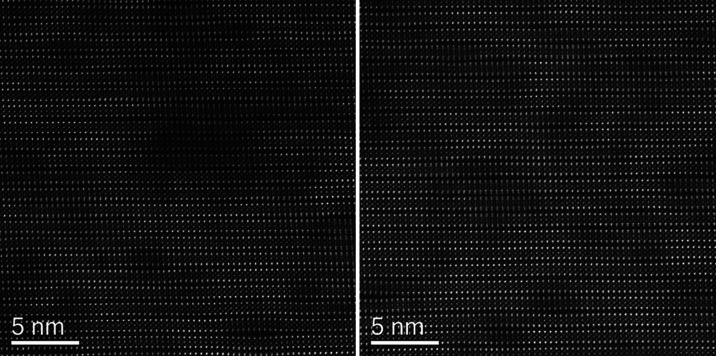
HAADF-STEM view of different areas of YBCO+ND film bulk. Short stacking faults are visible (partially hidden due to the sample thickness).

**Figure 6:**
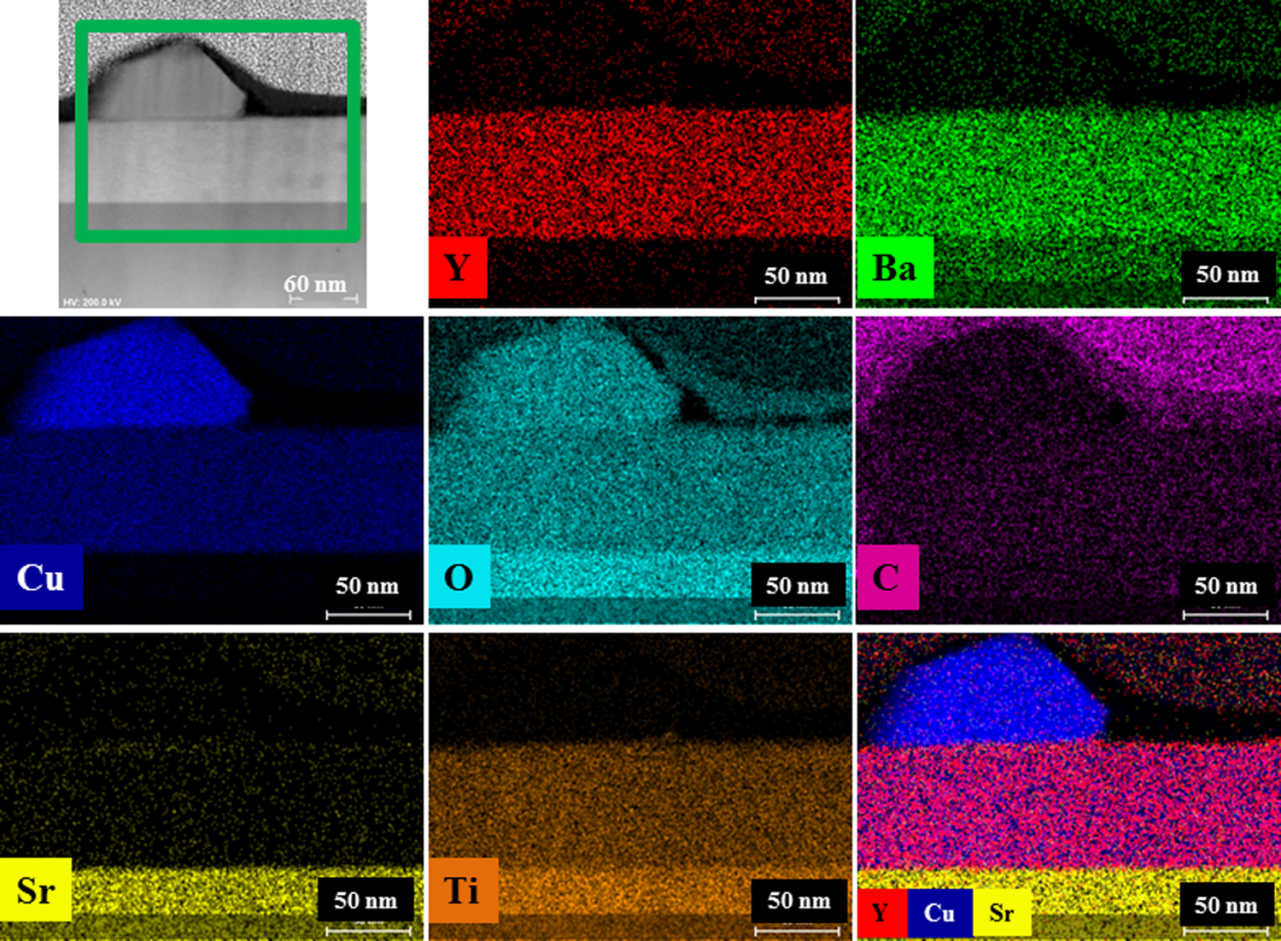
Cross-sectional view of the YBCO+ND film. The HAADF-STEM image showing the mapped area, evidenced by a green rectangle, together with elemental Y, Ba, Cu, O, C, Sr, and Ti EDX maps.

The YBCO layer grows epitaxially on the STO substrate: [010]STO//[100]/[010]YBCO ⟨001⟩STO//⟨001⟩YBCO. The interface with the substrate is very sharp and no nanoparticles or secondary phases could be distinguished (Figure 4). A large number of staking faults were observed at the outer surface, whereas this kind of defect was almost absent at the interface. Some misfit dislocations were observed on the STO–YBCO interface (lower panel of Figure 4). The film contains a number of short stacking faults (Figure 5). However, some secondary phases and outgrowths were observed. The STEM-EDX analysis provided information on the chemical composition of the film (Figure 6). The distribution of Y, Ba, Cu, and C in the whole analyzed zone is uniform, whereas at the surface, an accumulation of copper oxide is sometimes evident. No traces of ND particles were revealed, proving that they likely did not survive the thermal treatment.

**Figure 7:**
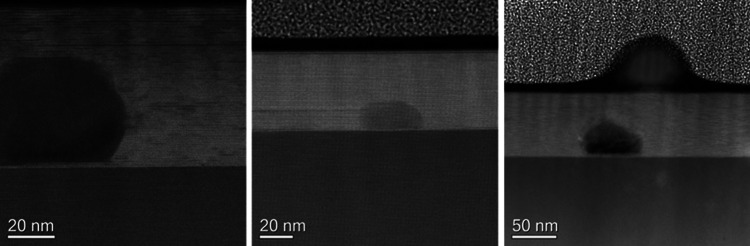
TEM cross-view of several regions evidencing the presence of unidentified defects.

The presence of several features at the interface is worth noticing, whose nature was not completely clarified by the EDX analysis but, most likely, these are holes (Figures 3b and 7). In particular, they were observed only at the interface and never embedded in the film matrix. The presence of this feature did not produce any increase of strain or other defects, e.g., stacking faults, in its surroundings as, on the contrary, revealed in films where other NPs are at the substrate interface and strain or stacking faults are evident in the film grown around the NP.^10,27−29^

The superconducting properties of fully converted YBCO+ND film confirmed the good results previously emerged. A sharp superconducting transition was observed, with a *T*_c_ value as high as 90.7 K, in line with the mean value^a^ of 90.4 ± 0.2 K measured with other YBCO+ND samples and similar to the best results achieved by YBCO films grown in the same conditions^a^ (*T*_c_ = 90.2 ± 0.4 K).^6^

Again, the film deposited with the solution containing ND showed an improvement of transport properties in self-field conditions that, as already hypothesized, could be originated from the excellent grain coalescence and film density, which promotes the current percolation path.^3^

### Nanodiamond Effect on YBCO Nucleation and Growth

**Figure 8:**
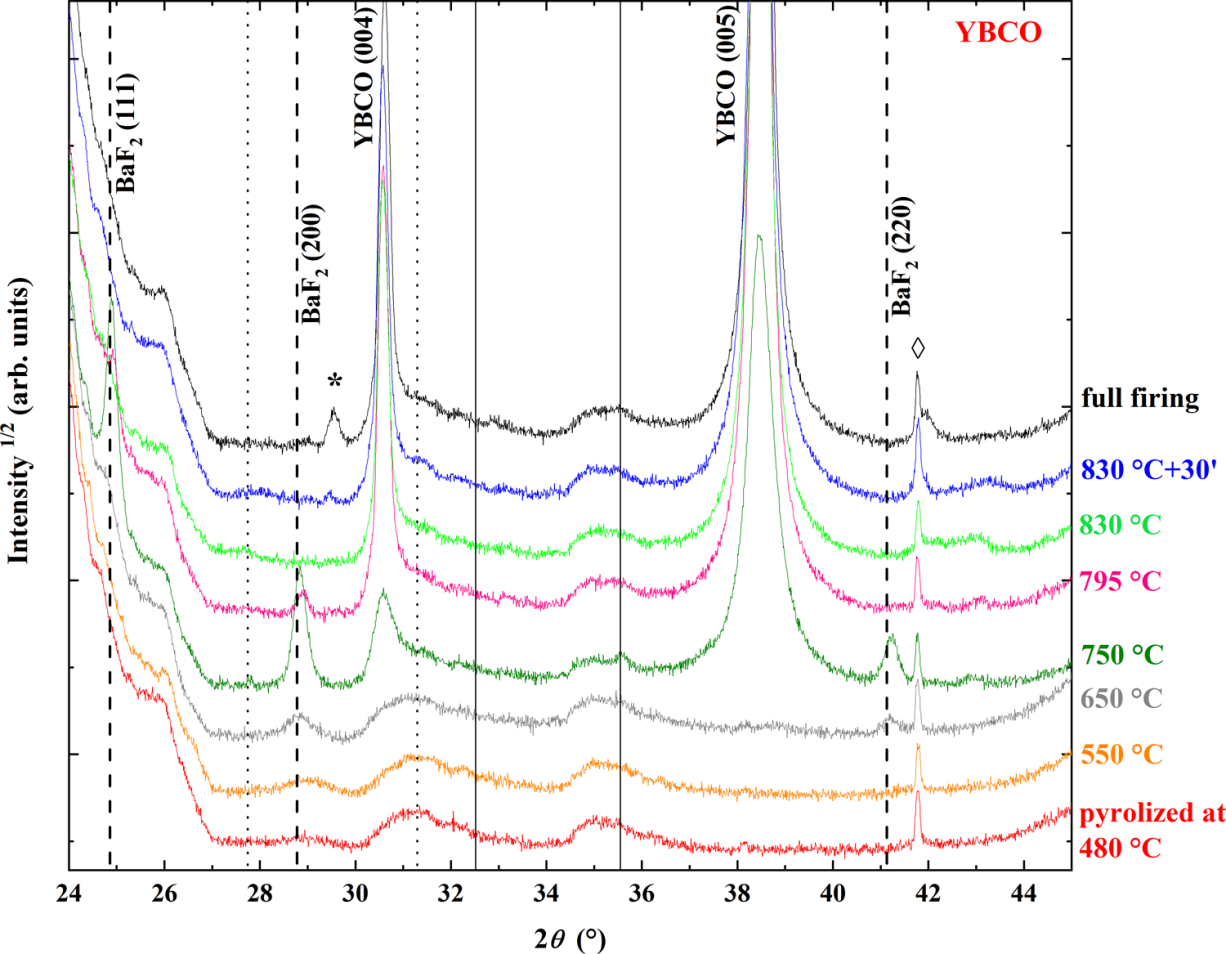
XRD θ–2θ patterns of partially converted YBCO samples: one sample pyrolyzed at 480 °C and six samples quenched after pyrolysis at different temperatures during the firing treatment. The curve named “830 °C + 30′” indicates a sample treated at 830 °C for 30 min before quenching. The pattern of a film subjected to the whole firing process and labeled as “full firing” is plotted for comparison. Vertical lines show the peak positions of BaF_2_ (dashed line), Y_2_Cu_2_O_5_ (dotted line), and CuO (continuous line). The labels of (111), (200), (220) BaF_2_ and (004), (005) YBCO peaks are also reported. The asterisk (*) evidences a Y–Ti–O secondary phase due to the solution–substrate interaction.^36,37^ The symbol (◊) marks the STO *K*_β_ peak.

The analysis of the XRD pattern of partially converted YBCO samples evidenced that, after the pyrolysis process, the precursor film was composed of Y_2_O_3_, CuO, and Ba_1–*x*_Y_*x*_F_2+*x*_, (BYF), the latter being a solid solution of YF_3_ in BaF_2_ with a fluorite crystalline structure (Figure 8). The observed conversion is in agreement with the study performed by Armenio et al.,^30^ in which a similar low-fluorine propionate solution was pyrolyzed using a comparable thermal treatment.

In Figure 8, XRD θ–2θ patterns of samples treated at different temperatures are plotted. Increasing the treatment temperature, relevant changes were observed. BYF peak intensity increased up to 750 °C and their position shifted first to lower angles. Yttrium fluoride exhibits solubility in the BaF_2_ fluorite crystalline structure in a wide range of compositions, *x*, forming a solid solution Ba_1–*x*_Y_*x*_F_2+*x*_. The increase of the YF_3_ concentration in the BaF_2_ lattice induces a linear elongation of the lattice parameter.^31^ Thus, a shift in the XRD BYF peak positions can be considered a signature of the Y composition variation. At 650 °C, the peak at 2θ = 28.8° can be attributed to pure BaF_2_, demonstrating the reduction of the Y fraction in the solid solution with increasing temperature. Then, YF_3_ conversion to Y-oxyfluorides and Y_2_O_3_ can be hypothesized in analogy with what was demonstrated by the XPS analyses reported in ref (30). At 750 °C, a second shift was observed at higher angles. This shift was previously attributed to Ba-oxyfluoride, BaF_2(1–*x*)_O_*x*_, (OF) formation.^32^ At the same time, evident (00) reflections of YBCO appeared at 750 °C in agreement with ref (30). However, in ref (30), at 750 °C, the peak intensities of OF (200) and YBCO (005) were comparable, while in the present work, at the same temperature, a sharp decrease of OF occurred and the OF peaks were negligible in comparison with YBCO peaks. This difference could be ascribed both to precursor solution concentration (more diluted in the present work and corresponding to a thinner film, namely, 0.2 M with respect to 0.4 M used by ref (30)) and to oxygen partial pressure used during the crystallization step, in our case lower than that in ref (30), i.e., O_2_/N_2_ is equal to 4 × 10^–4^ and 3 × 10^–2^, respectively. The effect of oxygen content in the atmosphere of heat treatment has been extensively studied and has significant importance on the process.^33−35^

Finally, a linear increase in the intensity of the YBCO phase was observed between the nucleation temperature and plateau temperature (830 °C). The YBCO peaks of the fully converted sample are slightly shifted toward higher angles due to the YBCO conversion from the tetragonal to orthorhombic phase occurring during the oxygenation treatment and subsequent to the crystallization step (Figure 1). Interestingly, the Y–Ti–O secondary phase appeared in the sample quenched after 30 min at 830 °C and was evident in the fully processed sample. Its origin is due to the solution–substrate interaction.^36,37^

In conclusion, considering these results and the literature data,^30,35,38^ these XRD results are consistent with the crystallization of YBCO through the chemical reaction I already proposed for fluorine-based MOD processes by several articles.^30,35,39−42^
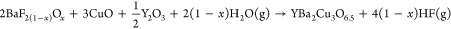
I

**Figure 9:**
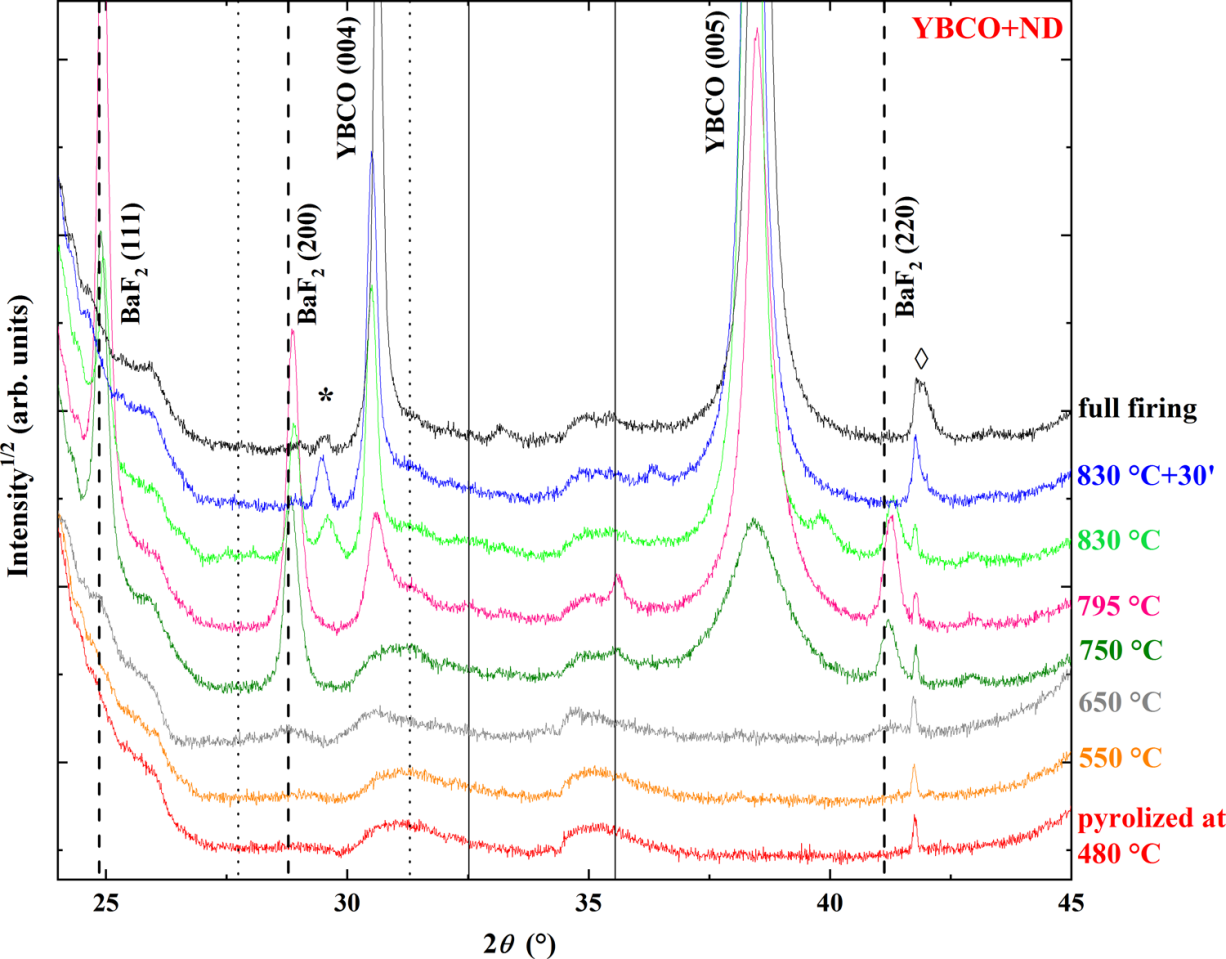
XRD θ–2θ patterns of partially converted YBCO+ND samples: one sample pyrolyzed at 480 °C and six samples quenched after pyrolysis at different temperatures during the firing treatment. The curve named “830 °C + 30′” indicates a sample treated at 830 °C for 30 min before quenching. The pattern of a film subjected to the whole firing process and labeled as “full firing” is plotted for comparison. Vertical lines show the peak positions of BaF_2_ (dashed line), Y_2_Cu_2_O_5_ (dotted line), and CuO (continuous line). The labels of (111), (200), (220) BaF_2_ and (004), (005) YBCO peaks are also reported. The asterisk (*) evidences a Y–Ti–O secondary phase due to the solution–substrate interaction.^36,37^ The symbol (◊) marks the STO *K*_β_ peak.

A similar study was then performed on the YBCO+ND system. In Figure 9, θ–2θ XRD spectra evidenced some interesting differences.

The evolution of BYF peaks was comparable to the YBCO film as regards peak shift, at first, to lower angles of up to 650 °C, evidencing the reduction of the Y fraction in the BYF phase. At 650 °C, the spectra of YBCO and YBCO+ND were very similar. By increasing the temperature in the YBCO+ND film, a further shift at higher angles was again revealed, indicative of OF formation. Similarly to the undoped sample, (00) YBCO reflections emerged. However, the intensity ratio of OF, considering the (111), (200), and (220) peaks and YBCO (005) significantly changed among YBCO and YBCO+ND films. In the latter case, at 750 °C a greater amount of OF was still evident with respect to the YBCO sample.

**Figure 10:**
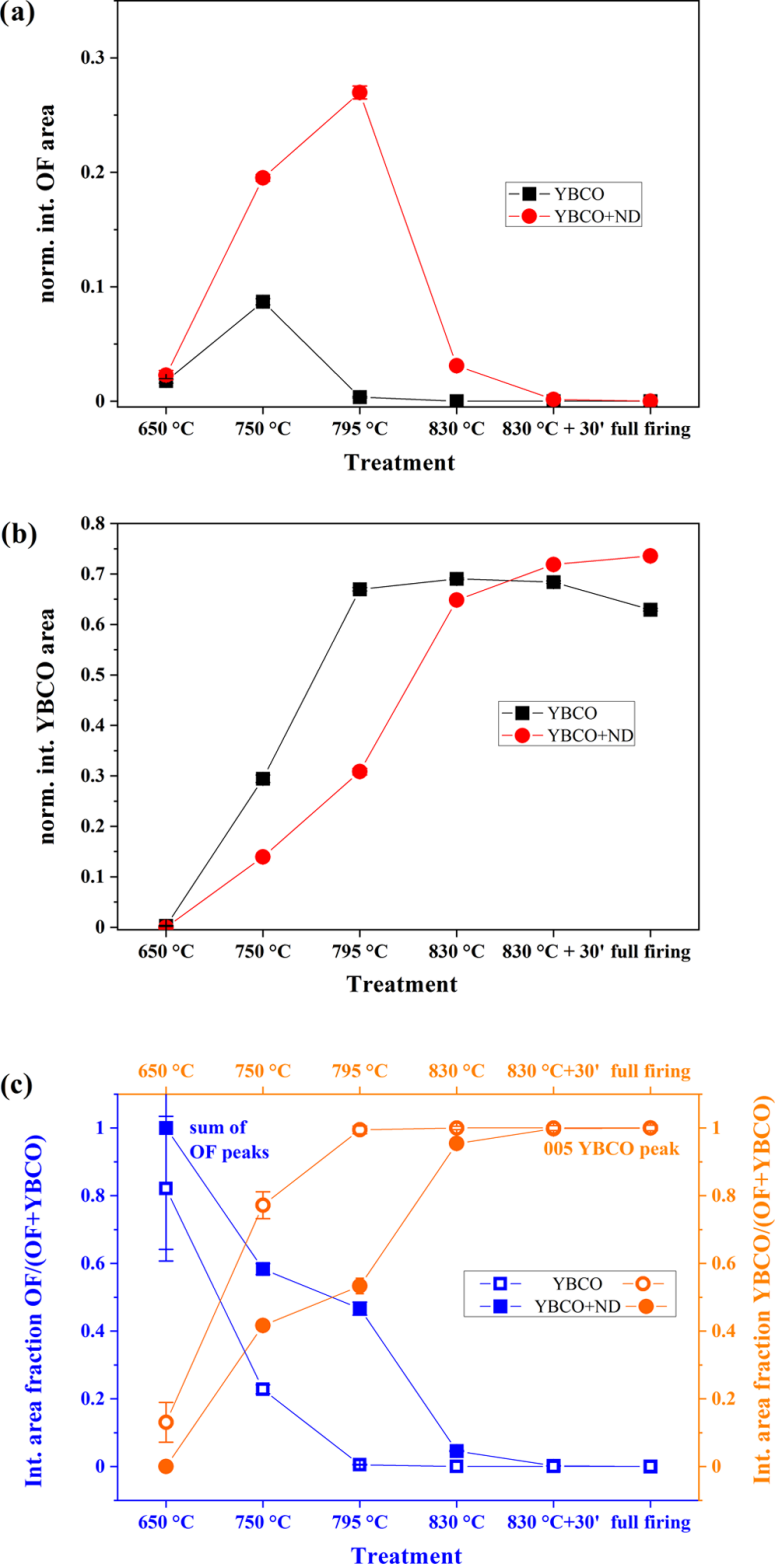
Temperature dependence of the XRD integrated intensity of (a) the sum of (111), (200), and (220) OF peaks (*I*^OF^) and (b) (005) YBCO peak (*I*^YBCO^) detected in YBCO (black squares) and YBCO+ND (red circles) precursor films after quenching during the conversion thermal treatment. Each peak was normalized to the total spectrum integral. (c) The OF (open and full blue squares) and YBCO (open and full orange circles) peak integrated area fractions, calculated according to  and , are reported for YBCO (open symbols) and YBCO+ND (full symbols), respectively. The error bars are, in most cases, smaller than the size of the symbols. The labels “830 °C+30′ ” and “full firing” refer to data obtained for the sample treated at 830 °C for 30 min before quenching and the fully converted film, respectively. The heating rate, up to 830 °C, was set equal to 30 °C·min^–1^ (Figure 1).

Even at 795 °C, a higher OF content was detected in relation to the YBCO film, where, on the contrary, OF peaks were negligible. The presence of ND seems to slow down reaction I to the point that after a 30 min plateau at 830 °C, OF in the YBCO+ND sample was still present. The evolution of integrated peaks, normalized to the total spectrum area, confirms this outcome. The integrated areas of OF peaks, considering the sum of (111), (200), and (220) peaks and the (005) YBCO peak, indicated as *I*^OF^ and *I*^YBCO^, respectively, detected in precursor films after quenching are reported in Figure 10a,b and Table 1. The comparison between YBCO and YBCO+ND film pairs evidenced how markedly ND affected reaction I in the temperature range of 750–830 °C. The integrated area fractions of OF and YBCO peaks were also calculated according to  and , respectively (Figure 10c and Table 1).

**Table 1:** Temperature Dependence of the XRD Normalized Integrated Area (Upper Panel) and Integrated Area Fraction (Lower Panel) of the Sum of (111), (200), and (220) OF (*I*^OF^) and (005) YBCO Peaks (*I*^YBCO^) Detected in YBCO and YBCO+ND Precursor Films after Quenching during the Conversion Thermal Treatment^a^

	normalized integrated area^b^			
thermal treatment	*I*^OF^		*I*^YBCO^	
	YBCO	YBCO+ND	YBCO	YBCO+ND
quench @650 °C	0.018 ± 0.002	0.022 ± 0.004	0.003 ± 0.001	
quench @750 °C	0.087 ± 0.003	0.195 ± 0.003	0.294 ± 0.007	0.139 ± 0.001
quench @795 °C	0.004 ± 0.001	0.270 ± 0.006	0.670 ± 0.003	0.308 ± 0.006
quench @830 °C		0.031 ± 0.001	0.690 ± 0.002	0.648 ± <0.001
quench @830 °C+30′		0.002 ± <0.001	0.684 ± 0.002	0.719 ± <0.001
full firing			0.629 ± 0.003	0.736 ± 0.001

	integrated area fraction^b^			
thermal treatment				
	YBCO	YBCO+ND	YBCO	YBCO+ND
quench @650 °C	0.821 ± 0.213	1.000 ± 0.359	0.131 ± 0.058	
quench @750 °C	0.228 ± 0.013	0.583 ± 0.016	0.772 ± 0.040	0.416 ± 0.008
quench @795 °C	0.005 ± 0.001	0.467 ± 0.019	0.995 ± 0.011	0.533 ± 0.023
quench @830 °C		0.046 ± 0.002	1.000 ± 0.005	0.954 ± 0.002
quench @830 °C+30′		0.002 ± <0.001	1.000 ± 0.007	0.998 ± 0.001
full firing			1.000 ± 0.008	1.000 ± 0.004

a Each peak was normalized to the total spectrum integral. The integrated area fractions of OF and YBCO peaks are calculated according to  and , respectively, and reported for YBCO and YBCO+ND, respectively. The labels “830 °C+30′ ” and “full firing” refer to data obtained for the sample treated at 830 °C for 30 min before quenching and the fully converted film, respectively.

b The integrated area of each peak was obtained from three different area measurements. The errors reported in the table were calculated according to the propagation of uncertainty formulas.

The intensities of the XRD spectra shown in Figures 8 and 9 provide qualitative insights into the evolution of the film composition during the heat treatment. To gain more quantitative information on the kinetics of film formation, we performed a simple comparative analysis of the data of Table 1, where the quenched samples are representative of the composition of the film at the time of quenching (i.e., at a temperature just before quenching).

The XRD intensity is proportional to the square of the number of unit cells of the compound, in the sampled volume, through the structure factor. It follows that the relative variation of intensity, in time interval Δ*t* = Δ*T*/ϕ (with ϕ = constant heating rate), is linked to the relative variation of the amount of the phase: , that is the rate of variation of the relative amount of compounds: . In the differential form, this relation implies , at constant ϕ.

**Figure 11:**
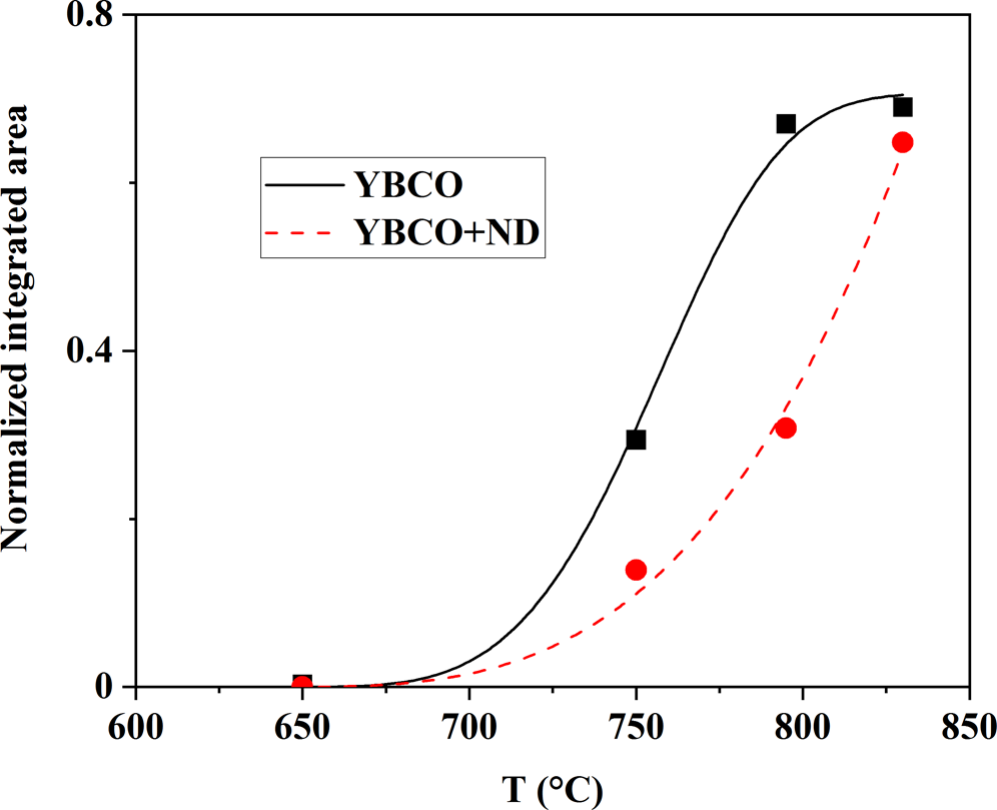
Experimental data of Table 1 fitted by continuous functions. Symbols show the temperature dependence of the XRD normalized integrated area of (005) YBCO peaks (*I*^YBCO^) detected in YBCO (black square) and YBCO+ND (red circle) precursor films after quenching during the conversion thermal treatment. Each peak was normalized to the total spectrum integral. The fit is represented by black continuous and red dashed curves for YBCO and YBCO+ND, respectively.

**Figure 12:**
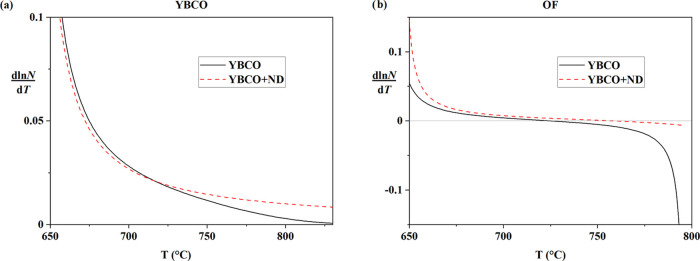
Comparison between the derivatives  (i.e., ) of YBCO (a) and OF (b) in both YBCO (black continuous curve) and YBCO+ND (red dashed curve) samples.

A rough computation of the kinetics of the relative amount of OF and YBCO phases can be attempted by considering OF intensity peaks due to a compound (i.e., assign a single structure factor to the peaks) and similarly for YBCO. To this end, the data points in Table 1 were fitted by a continuous (and derivable) function. The results of this approach are shown in Figure 11 for the YBCO e YBCO+ND data. The comparison between the derivatives  (i.e., ) of YBCO and OF in both YBCO and YBCO+ND is displayed in Figure 12a,b. The curves in Figure 12a indicate that at high temperatures (*T* > 730 °C), the relative variation of the amount of YBCO in YBCO+ND is greater than that of YBCO in YBCO. The relative variation of the amount of OF in YBCO+ND is greater than that of OF in YBCO in the whole temperature domain (Figure 12b).

This analysis, although based on few data points, gives more quantitative support to the evolution of the film composition derived in Figure 10. In particular, the kinetics of OF conversion into YBCO is slowed down in YBCO+ND compared to that in the YBCO sample, and this justifies a more extended reaction for YBCO formation at *T* > 730 °C. A crude interpretation of these behaviors can be proposed as follows.

Let us consider the first-order kinetics  and  for the formation and consumption of a compound with *K* and *K*′ effective rate constants (positive definite), respectively. Accordingly,  is equal to either *K*(*T*) or −*K*′(*T*). On this basis, the plots above indicate that the rate constant for YBCO formation in YBCO+ND is greater than that for YBCO formation in YBCO for *T* > 730 °C. As far as OF is concerned, the plot in Figure 12b shows that for *T* > 730 °C, OF is consumed in the YBCO sample, while in YBCO+ND, it is still under formation. It follows that the kinetics of OF production/conversion into YBCO is slowed down in YBCO+ND compared to that in the YBCO sample, and this entails a more extended reaction for the YBCO formation in the temperature domain *T* > 730 °C.

**Figure 13:**
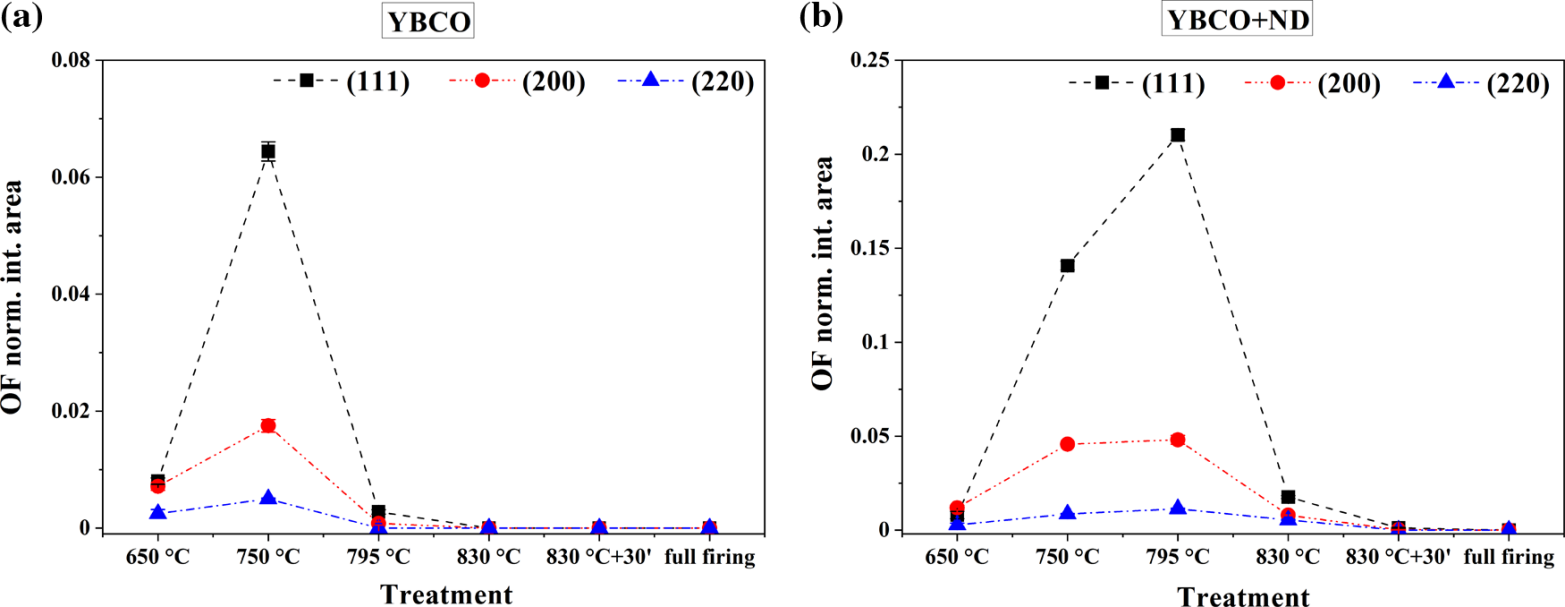
Temperature dependence of the XRD integrated intensity of (111), (200), and (220) OF (*I*^OF^) detected in YBCO (a) and YBCO+ND (b) precursor films after quenching during the conversion thermal treatment. Each peak was normalized to the total spectrum integral. The error bars are, in most cases, smaller than the symbol size. The labels “830 °C+30′ ” and “full firing” refer to data obtained for the sample treated at 830 °C for 30 min before quenching and the fully converted film, respectively.

The trend of the different OF growth directions has been also evaluated and does not change if ND is present. As can be seen in Figure 13, ND slows down the decomposition of the three phases in the same way and the integrated area of the (111) phase is the most intense one in both films.

Moreover, the signal of crystalline CuO was detected in the range of 750–795 °C in the YBCO+ND sample, while it was slightly visible only at 750 °C in the pure YBCO film. The more gradual CuO consumption could support the idea of a slower YBCO crystallization process.

Finally, the Y–Ti–O secondary phase was revealed in the samples quenched at 830 °C and fully converted (Figure 9). No trend of peak intensity could emerge, confirming that the formation of this phase randomly occurs and depends on substrate variability.^36,37^

## Discussion

Although the role played by ND is not fully discerned, some hypotheses can be formulated. In the literature, many papers have been devoted to nanocarbon fluorination and have shown that carbon of the CBN surface can easily react with fluorine to form a strong −C–F bond. Fluorination is considered one of the most effective chemical methods to modify and control the physicochemical properties of carbon materials.^43−49^ For example, Liu et al. reported the treatment of ND powder (characterized by a particle size ranging from 3.5 to 6.5 nm) with HF generated in situ.^46^ The authors stated that fluorination took place at a temperature above 150 °C and was easily obtained without any ND pretreatment, i.e., by hydrogenation or oxidation. The reaction was supposed to involve the already-existing surface functionalities, which have been activated by the in situ-generated HF catalyst. The IR spectrum showed that at 150 °C, all of the surface C–H bonds were completely fluorinated. By increasing the temperature to 310 °C, almost all C=O, C=C, and OH groups were removed or transformed. Increasing the fluorination temperature to 410 and 470 °C did not cause any significant changes in the IR spectra of the resulting fluoro-nanodiamond samples. Many other works reported fluorination reactions carried out in similar conditions^43,44,47,50^ that did not substantially differ from our situation in which F was present in the metal precursor, and HF was one of the reaction products (I) during the thermal treatment. Moreover, the C–F bond is particularly strong as demonstrated by several theoretical and experimental studies.^43,44,47,51,52^ The F-terminated ND surface is more stable and chemically inert than the H-terminated one. Therefore, fluorinated diamond is exceptionally resistant to thermal effects.^47^ In this sense, the work made by Ando et al. on fluorinated diamond powder is worth mentioning.^43^ Fluorine desorption, carried out using He as the carrier gas, started above 450 °C with distinctive desorption maxima at ∼600 and ∼1100 °C (see Figure 3 in ref (43)). Similarly, Smentkowski and Yates^52^ stated that surface-fluorinated diamond thermally decomposed in vacuum in the range of 900–1500 K, corresponding to approximately 630–1130 °C (Figure 4 in ref (52)). However, the fluorine desorption temperature range strongly depends on the considered diamond form (powder, nanopowder, crystal, film) and on the atmosphere.^44^

Actually, in our case, the ND fate was still not completely understood. TEM analyses showed a homogeneous but not relevant C distribution in the film matrix because no traces of ND particles or aggregates appeared. Therefore, ND was probably transformed, but at the moment we ignore the route of ND consumption. However, it is likely promoted by oxygen in the process atmosphere. Indeed, high temperatures are able to induce surface graphitization and subsequent combustion.^53,54^ Anyway, it should be considered that fluorination could be rather straightforward also taking into account other CBNs (CNT, graphene, onion-like structure).^49^ Therefore, during YBCO deposition, ND or its derivatives could interact with fluorine existing in the film matrix through both hydrogen and covalent bonds, the latter replacing its surface H or O atoms (present as C=O or C–OH). This reaction could be catalyzed by the HF produced during YBCO precursor decomposition.^55^ Hence, it should be presumed that strong C–F bond formation occurred and was probably resistant to our thermal treatment. YBCO nucleation was in some way influenced by this side reaction, despite the ND content in the precursor matrix being negligible with respect to the BaF_2_ amount. But, as for catalysis, only a small ND amount could be necessary. For example, fluorinated ND could act as a nucleation seed favoring OF coarsening, which would subsequently require a prolonged decomposition time. In other words, ND would have the effect of stabilizing the OF phase by making it kinetically less reactive. Otherwise, it could hamper the evolution of BaF_2_ toward BaF_2(1–*x*)_O_*x*_ (with *x* ≠ 0). As already evidenced by ref (56), the *x* value strongly affects the free energy change of reaction I; thus, at 750 °C and , a reduction of the *x* value corresponds to a critical increase of reaction free energy, Δ_r_*G*, (Figure 2 in ref (56)). Conversely, at 800 °C and , reaction I is spontaneous in the full *x* range. In any case, due to the presence of ND, we observed a delay of YBCO nucleation, which occurred at a higher *T* than usual, when the C–F bond could be more easily broken and/or reaction I could be favored from a thermodynamic point of view.

**Figure 14:**
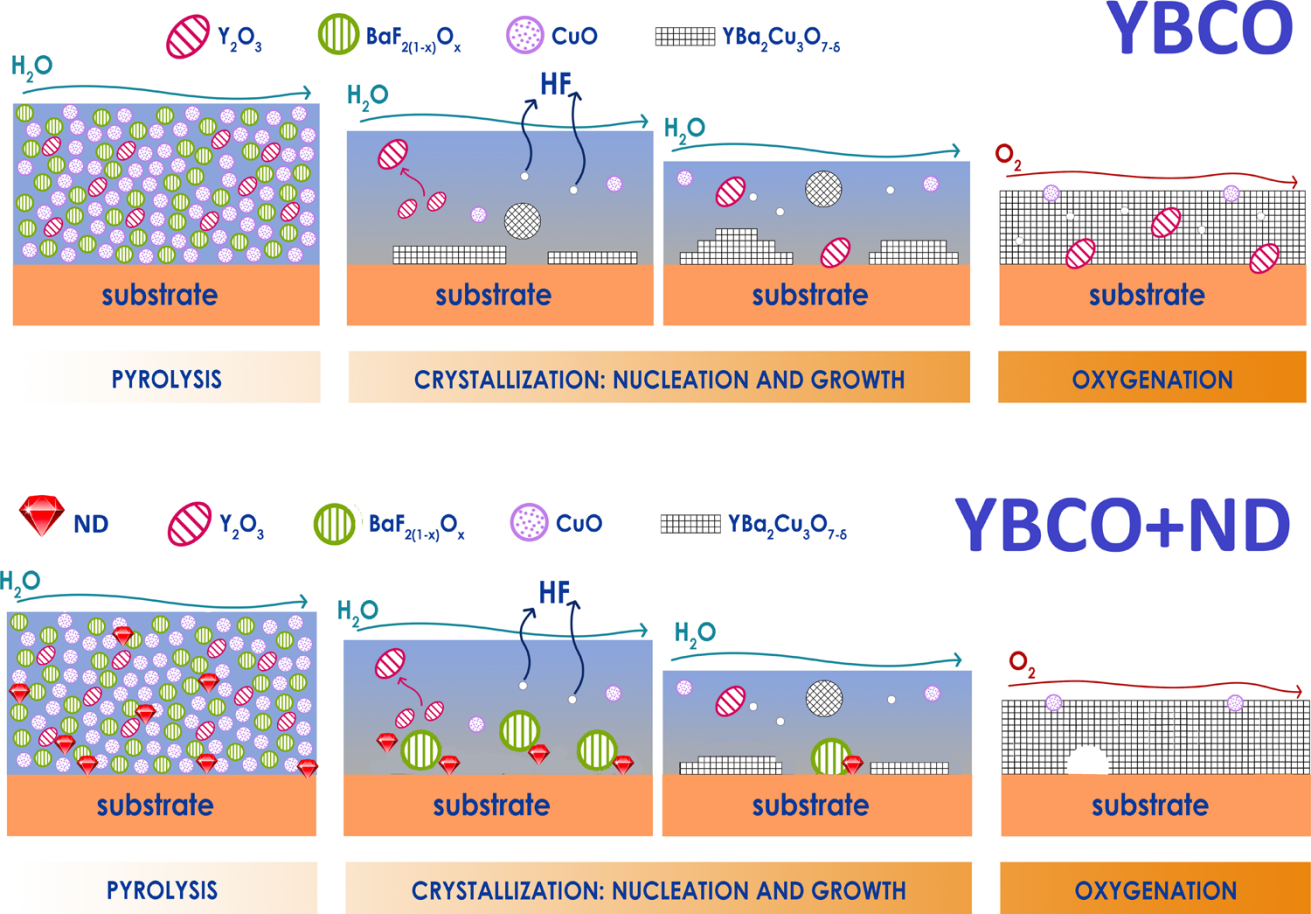
Schematic representation of the YBCO low-fluorine MOD process without and with ND.

However, regardless of the reaction mechanism, the excellent morphology of YBCO+ND with improved superconducting properties should derive from the possibility of starting nucleating at higher temperatures, when the best conditions for *c*-axis nucleation occur.^56^ A schematic representation of ND influence on the YBCO deposition process is reported in Figure 14.

Certainly, our hypotheses should be verified by further investigation. But, at this stage, these results could have interesting perspectives. For example, the effect of ND on YBCO nucleation could represent a route to directly reach the ideal temperature window for *c*-axis nucleation as similarly proposed and recently obtained by the “ultrafast transient liquid assisted growth” and “flash heating” procedures.^9,15,57,58^ These approaches used very fast heating rates (up to 80 °C·s^–1^) to deposit highly *c*-axis YBCO films by quickly crossing the temperature region where undesired phases could nucleate.

## Conclusions

In this paper, the study of the influence of ND on YBCO nucleation mechanisms has been reported. Specifically, in YBCO+ND samples, the chemical reaction responsible for YBCO nucleation was inhibited and occurred at higher temperatures compared to pristine samples. Further investigations will be necessary in order to evaluate the exact ND role. However, a possible scenario was proposed. Anyway, the increased nucleation temperature promoted *c*-axis crystallization, with consequent formation of highly epitaxial films.

As regards ND fate, TEM analyses did not evidence any ND particle in the film matrix. Probably, ND did not survive the used thermal treatment. However, its beneficial effect on YBCO nucleation can be further exploited and the opportunity to use ND in combination with other conventional APCs will be evaluated.

## Abbreviations

ADF-STEM: annular dark-field scanning transmission electron microscopy
APCs: artificial pinning centers
CBNs: carbon-based nanomaterials
CSD: chemical solution deposition
EDX: energy-dispersive X-ray spectroscopy
FWHM: Full width at half-maximum
HAADF-STEM: high-angle annular dark-field scanning transmission electron microscopy
*J*_c_: critical current density
MOD: metal–organic decomposition
ND: nanodiamond
NPs: nanoparticles
OF: BaF_2(1–*x*)_O_*x*_
PLD: pulsed laser deposition
*RE*BCO: *RE*Ba_2_Cu_3_O_7–x_, in which *RE* is Y or rare earth
SEM: scanning electron microscopy
STO: SrTiO_3_
*T*_c_: critical temperature
TEM: transmission electron microscopy
YBCO: YBa_2_Cu_3_O_7−δ_
XRD: X-ray diffraction

## Footnotes

a The mean value is expressed as the mean and standard deviation of results obtained for several samples prepared in the same laboratory and with the same procedure
